# Breaking new ground in research

**Published:** 2009-12

**Authors:** AJ Brink

**Affiliations:** Editor-in-Chief

A recent publication on the identification of the gene for progressive familial heart block (PFHB) type 1 in the *Journal of Clinical Investigation*^1^ illustrates various values in the successful pursuit of new knowledge. The condition, first described with a detailed genealogy of the family concerned,^2^ was rewarded with a prize for the best article in the *South African Medical Journal* in 1977. Furthermore, it gave rise to several articles by other authors, on the extent of the disease^3^ in a follow-up study after 10 years^4^ (and to a thesis on possible clinical characteristics of identification in family members^5^). Brink and Corfield continued research on this condition on a molecular genetic basis over the ensuing years at the Centre for Molecular and Cellular Research at the University of Stellenbosch.

The disease affects the electrical system of the heart as opposed to diseases that affect the blood vessels, such as coronary artery disease. Heart block causes palpitations, light-headedness, fainting and sudden, unexpected death. This condition can present at any age and, as in the original proband first studied, even in utero. Management depends on the insertion of a pacemaker at the appropriate time.

In 1986, the probable locus for the condition was narrowed down to an area encompassing about 80 genes on chromosome 19, which contains just over 1 500 genes.^6^ The identification of the gene was close to impossible at that time with local resources and existing methodologies.

About the same time, Prof Olaf Pongs of Hamburg University’s Centre for Molecular Neurobiology was researching a link between the gene TRPM4 on the 19th chromosome and PFHB type 1. The subjects of his study were all Afrikaans-speaking South Africans. The two research groups decided to join hands and this joint effort eventually led to the discovery of the gene for PFHB type 1.

It became clear that the product of this gene was playing a role in the way heart cells handle sodium and potassium, which underlies the electrical signals of the heart. The discovery was a breakthrough for South African medical science as it gave hope to sufferers who can now get advance warning of their condition and receive the necessary treatment.

Persistent research over a 35-year period spanning two generations of researchers (father and son: Andries and Paul Brink), and making use of new opportunities and international co-operation has contributed to breaking new ground and advancing the field of medicine.
